# Dietary fat content and supplementation with sodium butyrate: effects on growth performance, carcass traits, meat quality, and myopathies in broiler chickens

**DOI:** 10.1016/j.psj.2024.104199

**Published:** 2024-08-08

**Authors:** A. Huerta, G. Xiccato, F. Bordignon, M. Birolo, M. Boskovic Cabrol, F. Pirrone, A. Trocino

**Affiliations:** ⁎ Department of Agronomy, Food, Natural Resources, Animals and Environment (DAFNAE), University of Padova, 35020 Legnaro, Padova, Italy; † Department of Comparative Biomedicine and Food Science (BCA), University of Padova, 35020 Legnaro, Padova, Italy

**Keywords:** sodium-butyrate, dietary energy, sex, growth performance, carcass trait

## Abstract

This study aimed to evaluate the effects of the dietary inclusion of microencapsulated sodium butyrate (Na-butyrate; 0, 150, and 300 mg Na-butyrate/kg diet) and dietary fat reduction (7.7% vs. 6.7% in the grower diet; 8.9% vs. 7.7% in the finisher diet) in 792 (half male and half female) broiler chickens on growth performance, carcass traits, and meat quality and the occurrence of wooden breast (**WB**), white striping (**WS**), and spaghetti meat (**SM**). Dietary supplementation with Na-butyrate did not affect the growth performance, carcass traits, meat quality traits, or myopathy rates. Dietary fat reduction did not influence feed intake (**FI**) but decreased average daily gain (**ADG**); increased feed conversion ratio (**FCR**) (*P* < 0.001); and decreased the occurrence of WS (–38%; *P* < 0.01), WB (–48%; *P* < 0.05), and SM (–90%; *P* < 0.01). Dietary fat reduction also increased cold carcass weight (*P* < 0.01), carcass yield (*P* < 0.05), and *pectoralis major* yield (*P* < 0.05), whereas meat quality was not affected. Compared to females, males had high body weight, ADG, and FI and low FCR (*P* < 0.001) at the end of the trial. Moreover, cold carcass weight and hind leg yield were higher in males than in females (*P* < 0.001), whereas females had higher carcass, breast, and *p. major* yields (*P* < 0.001). Males showed a higher rate of WB (*P* < 0.001) and a lower rate of SM (*P* < 0.01) than females, whereas WS occurrence did not differ between sexes. In conclusion, Na-butyrate supplementation did not affect growth performance, carcass traits, or meat quality. Conversely, the reduction in dietary fat greatly decreased myopathy occurrence, whereas moderately impaired growth performance.

## INTRODUCTION

The preference for chicken meat has doubled in the last 2 decades globally (+110% from 2000 to 2022) (FAOSTAT, 2024) because of the lack of religious or cultural restrictions, high nutrient content, environmental sustainability, and economic affordability (Wideman et al., 2016; Bailey, 2023). To meet this demand and corresponding production, the poultry industry has focused on the intensive selection of genotypes with fast growth and high breast yields (Havenstein et al., 2003; Bailey et al., 2015; Lee and Mienaltowski, 2023) supported by feeding programs that combine high dietary protein and energy levels. However, this enhanced performance has resulted in the appearance of meat defects in the breast, that is, myopathies, in addition to changes in the sensory properties of meat (texture, color, and flavor), with consequences for customer acceptance and purchase intentions (Mir et al., 2017) and significant economic losses for the poultry industry (Xing et al., 2020; Bordignon et al., 2022; Che et al., 2022). The effects of myopathies such as wooden breast (**WB**), white striping (**WS**), and spaghetti meat (**SM**) on the quality, nutritional value, and technological and functional properties of raw and cooked meat have been well documented (Mudalal et al., 2015; Baldi et al., 2018; Soglia et al., 2021). To minimize the occurrence of these muscle abnormalities, changes in growth trajectories have been tested using different strategies to shape how individual birds achieve their final body weight and breast yield over time (Bailey, 2023; Trocino et al., 2023). Among these strategies, qualitative (e.g., protein, amino acids, and energy) and quantitative feed restriction programs have been proposed (Trocino et al., 2015; Meloche et al., 2018b; Gratta et al., 2019), which can affect both performance and meat quality (Kuttappan et al., 2012a, b) to different extents depending on the degree and duration of the restriction.

In contrast, to further improve sustainability, the poultry industry is working toward the development of alternatives, including antibiotic-free production. In this context, different feed additives are attractive because of their beneficial effects on animal health and immunity (Caly et al., 2015; Ayalew et al., 2022). Short-chain fatty acids have been shown to be effective feed additives under antibiotic-free conditions (Leeson et al., 2005), wherein butyrate is rapidly used within the gastrointestinal tract of birds (Wu et al., 2018). Butyrate improves the development of the intestinal mucosa and morphological structures by increasing the growth and overall surface area of absorption of the villi and enhancing the absorption and metabolism of nutrients (Hu and Guo, 2007; Smulikowska et al., 2009; Guilloteau et al., 2010; Wu et al., 2016). It has notable bactericidal and bacteriostatic effects on pathogenic bacteria in the gut (Van Immerseel et al., 2004). Previous studies have shown that supplementation with protected forms of butyrate enhances animal performance and carcass yield (Leeson et al., 2005; Smulikowska et al., 2009; Bedford et al., 2017) while providing anti-inflammatory and immune-enhancing properties (Sunkara et al., 2011). Pascual et al. (2020) reported a reduction in the number of SM breasts in females supplemented with microencapsulated sodium butyrate (Na-butyrate). The available literature on the effects of Na-butyrate on meat quality is inconsistent. A few studies have shown that dietary inclusion of Na-butyrate results in darker meat and higher pH (Gao et al., 2022), a decrease in saturated fatty acid content in the breast meat of chickens exposed to stress (Zhang et al., 2011), whereas other authors did not report effect on meat quality traits (Pascual et al., 2020).

Thus, given the available information on the effects of strategies for modulating growth trajectory and butyrate supplementation in poultry, the present study evaluated the effect of a modest reduction in dietary energy by modulating fat content and dietary supplementation with Na-butyrate on the performance, myopathy occurrence, carcass traits, meat quality, and sensory properties of broiler chickens.

## MATERIALS AND METHODS

### Experimental Facilities

The trial was performed at the poultry house of the Experimental Farm “L. Toniolo” of the University of Padova (Legnaro, Padova, Italy), after 6 mo of downtime. The poultry house was equipped with a cooling system, forced ventilation, radiant heating, and a controlled light system. Thirty-six wire-net pens (2.5 × 1.2 m; 3 m^2^) with 1.2 m-high wire-net walls were available, each equipped with 5 nipple drinkers and a circular feeder for manual distribution of feed. Each pen had a concrete floor covered with 5-cm wood shavings and chopped wheat straw litter. A total of 24 h of light was provided for the first 2 d after the chickens arrived at the poultry house. The hours of light were then progressively reduced until an 18L:6D photoperiod was achieved, which was maintained from 13 d of age onwards. During the trial, temperature and relative humidity, recorded in the poultry house using a data logger (P5185, PeakTech, Prüf- und Messtechnik GmbH Gerstenstieg, Ahrensburg, Germany) placed in the center of the room at 30 cm above the ground, averaged at 24.6 ± 2.9°C and 48.0 ± 8.5%, respectively.

### Animals, Experimental Groups, and *In Vivo* Recordings

This study was approved by the Ethics Committee for Animal Experimentation of the University of Padova (Project 82/2022 - Prot. No. 246564, approved on 19/12/2022). All animals were handled according to the principles of the EU Directive 2010/63/EU on the protection of animals used for experimental and other specific purposes.

A total of 900 broiler chicks (1-day-old; 450 males and 450 females; Ross 308; Aviagen, Huntsville, AL) were delivered by commercial truck to the experimental facilities. All chicks were vaccinated against Marek's disease, infectious bronchitis, and avian pseudopestis at the hatchery. They were randomly allocated to 36 pens (25 birds per pen) according to a trifactorial arrangement with 12 experimental groups (with 3 pens and 75 birds per experimental group) obtained by the combination of 2 levels of fat (18 pens and 450 birds per level of fat) × 3 levels of dietary Na-butyrate supplementation (12 pens and 300 birds per level of butyrate) × 2 sexes (18 pens and 450 birds per sex) and controlled from the day of arrival until commercial slaughtering at 42 d of age. During the trial, 3 chickens per pen were slaughtered at 14, 28, and 40 d of age to sample the gut tissues and contents for histological examinations and microbiota analyses, respectively (data not reported in the present study). On the day of their arrival, the chicks were identified using a plastic band with a unique number on their legs and were individually weighed. They were weighed once a week until slaughter at 42 d of age using an electronic balance (Wunder, Sa.Bi. s.r.l., Milan, Italy). Food and water were provided *ad libitum*. Daily feed intake was recorded at the pen level using an automated computerized weighing system connected to all feeders. Mortality was assessed daily.

### Diets and Feeding Plans

Three diets were formulated for feeding during 3 periods: P1, starter diet, fed to all animals from the arrival of chickens (1-day-old) to 14 d of age; P2, grower diet, administered from 15 to 28 d of age, with 2 fat (ether extract, **EE**) levels (high fat-**HF** diet: 7.7% EE vs. low-fat-**LF** diet: 6.7% EE); and P3, finisher diet, fed from 29 to 42 d, with 2 fat levels (HF diet: 8.9% EE vs. LF diet: 7.7% EE) (Table 1). In each period and within each fat level, the treatment with Na-butyrate consisted of the following 3 diets: control diet (B0), control diet supplemented with 150 mg/kg of Na-butyrate (diet B150), and control diet supplemented with 300 mg/kg of Na-butyrate (diet B300). The control diets, HF and LF, were produced in crumble form using an industrial feed mill (Nuova Padana Mangimi, Piove di Sacco, PD, Italy). Commercial microencapsulated Na-butyrate (30% Na-butyrate) was then added to obtain the experimental diets at the experimental farm by thoroughly mixing the Na-butyrate product with diet B0 using an electric concrete mixer (Suncoo 4/5HP Concrete mixer, 140 L, 600 W; SUNCOO, China). In detail, 3 kg of the B0 diet was progressively added to the commercial additive and mixed by hand in a box prior to mixing with another 47 kg of diet in the electric concrete mixer to obtain the final diets B150 and B300. All diets were analyzed in the laboratory of the Department of Agronomy, Food, Natural Resources, Animals and Environment (**DAFNAE**) to determine their dry matter, ash, crude protein, and starch (amyloglucosidase-α-amylase method) levels using AOAC (2000) methods. The EE level was analyzed after acid hydrolysis (EC, 1998).

**Table 1:** Ingredients and chemical composition of the control diets (%).

Periods	Starter period (d 1–14)	Grower period (d 15–28)		Finisher period (d 29–42)	
Diets	B0	B0-H	B0-L	B0-H	B0-L
Ingredients, %					
Corn meal	56.10	56.18	59.13	56.25	62.15
Soybean meal 48% CP	35.0	31.0	29.8	27.0	24.6
Full fat soybean	3.00	6.00	5.50	9.00	8.00
Animal fat	2.50	3.75	2.50	5.00	2.50
Dicalcium phosphate	1.00	0.28	0.28	0.25	0.25
Calcium carbonate	0.92	1.15	1.15	1.30	1.30
Liquid methionine (40% L-methionine)	0.31	0.18	0.24	0.23	0.23
Sodium chloride	0.26	0.60	0.60	0.27	0.27
Vitamin mineral supplement^1^	0.25	0.26	0.26	0.25	0.25
Liquid lysine (50% L-lysine)	0.25	0.18	0.18	0.16	0.16
Phytase^2^	0.20	0.23	0.23	0.20	0.20
L-threonine	0.11	0.05	0.05	0.04	0.04
Biotin	0.05	0.08	0.08	0.05	0.05
Coccidiostat^3^	0.10	0.05	0.05	–	–
Nutrients, % as fed					
Dry matter	88.3	89.0	88.8	88.8	88.6
Crude protein	21.7	20.5	20.2	19.2	18.8
Crude fibre	1.43	1.47	1.65	1.79	1.88
Ether extract	6.19	7.67	6.68	8.90	7.65
Ash	5.16	4.80	4.85	4.60	4.61
Starch	33.7	33.4	35.7	33.8	37.1
Calcium^4^	0.80	0.76	0.76	0.72	0.71
Phosphorus^4^	0.58	0.50	0.50	0.42	0.42
Lysine^4^	1.40	1.33	1.29	1.26	1.17
Methionine + cysteine^4^	0.75	0.73	0.72	0.71	0.69
Threonine^4^	0.96	0.90	0.88	0.84	0.80
Apparent metabolizable energy^4^, kcal/kg	2999	3107	3056	3215	3113

Abbreviations: B0: Control diet without Na-butyrate supplementation; H, Higher dietary fat; L, Lower dietary fat; CP: Crude protein.

1 Premix provided per kg of feed: vit. A, 10,000 IU; vit. D_3_, 3500 IU; vit. E acetate, 90 mg; vit. K_3_, 6 mg; Biotin, 0.38 mg; Thiamine, 3.75 mg; Riboflavin, 8 mg; vit. B_6_, 5.75 mg; vit. B_12_, 0.04 mg; Niacin, 70 mg; Pantothenic acid, 17.5 mg; Folic acid, 2.25 mg; Fe, 45 mg; Cu, 10 mg; Mn, 70 mg; Zn, 65 mg; Se, 0.25 mg.

2 Ronozyme HiPhos (DSM - Firmenich AG, Kaiseraugst, Switzerland).

3 Sodium monensin, 100 mg/kg feed.

4 Values calculated according to De Blas et al. (2019).

### Commercial Slaughtering and Carcass and Meat Quality Assessment

At 42 d of age, all birds were slaughtered in a commercial slaughterhouse. The chickens were weighed individually before crating after 4 h of fasting. Loading took approximately 1 h, transport from the experimental facilities to the commercial slaughterhouse approximately 15 min, and lairage before slaughtering approximately 3 h. All ready-to-cook carcasses were recovered after 2 h of refrigeration at 2 °C and individually weighed to measure carcass yield (Working Group 5, World's Poultry Science Association, 1984). A total of 144 carcasses (4 carcasses per pen), previously selected based on the final live weight of chickens corresponding to the mean body weight within a pen, were subjected to gross examination to evaluate the occurrence (presence or absence) in the *pectoralis major* muscles for white striping (WS) (Kuttappan et al., 2012a), wooden breast (WB) (Sihvo et al., 2014), and spaghetti meat (SM) (Baldi et al., 2018). Then, 72 carcasses (2 per pen) were selected and transported to the DAFNAE laboratory and stored at 2 °C. Twenty-four h after slaughter, the carcasses were dissected for the main cuts (breasts, wings, thighs, and drumsticks), and *p. major* muscles were separated for meat quality analyses (Petracci and Baéza, 2011). A pH meter (Basic 20; Crison Instruments Sa, Carpi, Italy) equipped with a specific electrode (cat. 5232; Crison Instruments Sa) was used to measure the pH values in triplicate on the ventral side of the right *p. major* muscle. The color indices L*, a*, and b* were measured in triplicate at the same position using a Minolta CM-508 C spectrophotometer (Minolta Corp., Ramsey, NJ) (Petracci and Baéza, 2011). After measuring the pH and color indices, 1 meat portion (8 × 4 × 3 cm) was separated from the cranial side of the *p. major* muscle, parallel to the direction of the muscle fibers, and stored under vacuum in plastic bags at −18 °C until meat analyses. After thawing, each bag with its meat portion was cooked in a water bath at 80 °C for 40 min (Petracci and Baéza, 2011). After 40 min of cooling, a smaller meat portion (4 × 2 × 1 cm) was separated to assess the maximum shear force using an LS5 dynamometer (Lloyd Instruments Ltd., Bognor Regis, UK) with an Allo-Kramer (10 blades) probe (load cell, 500 kg; distance between the blades, 5 mm; thickness, 2 mm; cutting speed, 250 mm/min) (Mudalal et al., 2015).

### Meat Sensory Evaluation

As described by Huerta et al. (2023), to obtain the samples for tasting, the left *p. major* muscles of chickens were defrosted overnight at 4 °C. The breasts were cut in pieces (7 × 8 × 1.5 cm) and then cooked sealed in bags under vacuum in a water bath at a constant temperature, i.e., 85 °C for 25 min. Thereafter, each meat piece was divided into 4 equal samples (3 × 2 × 1 cm) and immediately served to a panel trained according to ISO standards (ISO 8586, 3972, 5496), consisting of 12 members of the DAFNAE laboratory (6 males and 6 females) aged between 23 and 60 yr.

Quantitative descriptive sensory analysis of the left *p. major* muscle was performed by the panel. The panelists evaluated the 11 descriptors on a structured continuous line scale with anchor points of 0 (not intense) and 10 (very intense). The assessors were asked to evaluate 1 chicken breast at a time, starting from the odor attributes, followed by the taste and texture attributes, and finally general liking (pleasantness). After each sample, the panelists cleaned their palate with a piece of apple, unsalted crackers, and mineral water and took a 2-min break, after which they continued with the next sample.

Four sessions were conducted over a period of 2 wk. For each daily session, 2 sets of 3 chicken breasts (each belonging to a different experimental group) were presented, and 15-min breaks were provided between the 2 sets. The presentation order was systematically varied using a Williams Latin square design to balance the effects of serving order and the carryover effect. Data were collected using Fizz v2.47b software (Biosystemes, Couternon, France).

### Statistical Analysis

Individual data on live weight, daily growth, slaughter yield, carcass dissection, and meat quality traits were subjected to an analysis of variance (**ANOVA**) with fat content, Na-butyrate supplementation, sex, and their interactions as the main factors of variability; the individual birds as the experimental unit; and the pen as a random effect, using the PROC MIXED procedure of SAS (SAS Institute, 2013) and the following model:

$$Y_{\text{ijkl}}=\mu +F_{i}+B_{j}+S_{k}+FB_{\text{ij}}+FS_{\text{ik}}+BS_{\text{jk}}+\text{FB}S_{\text{ijk}}+e_{\text{ijkl}}$$

where µ is an overall mean response, F represents the effect of the _i_ level of the fat level (high vs. low), B represents the effect of the _j_ level of the Na-butyrate inclusion (0, 150, 300 mg/kg), S represents the effect of the _k_ level of the sex (females vs. males), Y is the response of the experimental unit, and e is the observation error.

The same main factors were used to test the differences in pen data for feed intake and feed conversion using the PROC GLM procedure of SAS, with the pen as the experimental unit. Myopathy rates were analyzed using PROC CATMOD of SAS. Sensory results were analyzed using the PROC GLIMMIX of SAS with fat content, Na-butyrate supplementation, sex, and their interactions as fixed effects and the assessor as a random effect. A Poisson distribution was assumed for the data.

## RESULTS

No significant interactions were detected between the main experimental factors, for which the data are listed in the tables as the least square means of the main factors.

### Growth Performance

On the day of hatching, the average chick weight was 44.1 ± 3.8 g without significant differences between dietary treatments (Table 2). At 14 d of age, chickens supplemented with diet B150 had the highest live weight, whereas chickens fed diet B0 had the lowest live weight, with intermediate values for chickens fed the diet B300 (Table 2). At 42 d of age, chickens reached an average live weight of 3,009 g, and the dietary supplementation of Na-butyrate did not affect daily weight gain (on average 70.6 g/d), feed intake (105.8 g/d), or feed conversion ratio (1.51) throughout the trial (Table 2).

**Table 2:** Growth performance (LS means) from hatching until commercial slaughtering at 42 d of age of broiler chickens fed diets with different Na-butyrate supplementation and fat contents.

Variables	Na-butyrate (B)			Fat (F)		Sex (S)		*P*-value							RMSE
	B0	B150	B300	H	L	F	M	B	F	S	B × F	B × S	F × S	B × F × S	
Chickens, n	252	256	252	376	384	380	380								
Live weight 1 d,^1^ g	43.4	43.7	43.1	43.3	43.5	45.1	41.8	0.168	0.440	<0.001	0.560	0.378	0.597	0.654	3.41
Live weight 13 d,^1^ g	380^a^	389^b^	381^ab^	386	381	380	387	<0.05	0.158	<0.05	0.761	0.807	0.786	0.912	41.5
Live weight 27 d,^1^ g	1444	1455	1446	1458	1439	1379	1518	0.554	<0.05	<0.001	0.766	0.464	0.215	0.856	118
Live weight 42 d, g	3011	3006	3011	3046	2973	2770	3249	0.958	<0.001	<0.001	0.346	0.763	0.278	0.462	229
Daily weight gain,^1^ g/d	70.7	70.5	70.7	71.5	69.8	64.9	76.4	0.950	<0.001	<0.001	0.341	0.756	0.273	0.468	5.45
Feed intake,^2^ g/d	106	106	106	106	106	99.2	112	0.889	0.575	<0.001	0.538	0.959	0.582	0.612	1.59
Feed conversion^2^	1.50	1.51	1.51	1.48	1.53	1.53	1.48	0.502	<0.001	<0.001	0.498	0.508	0.052	0.133	0.018

Abbreviations: RMSE, root mean square error where SEM is equal to RMSE/√n; B0, Control diet; B150, control diet supplemented with 150 mg/kg of Na-butyrate; B300, control diet supplemented with 300 mg/kg of Na-butyrate; H, Higher dietary fat content; L, Lower dietary fat content; F, females; M, males.

1 Individual data.

2 Pen data.

In terms of dietary fat content, chickens fed the LF diet had a lower daily weight gain (−2.5%; *P* < 0.001), final live weight (−2.4%; *P* < 0.001), and a higher feed conversion ratio than chickens fed the HF diet (Table 2).

As speculated, males were heavier than females from the first day until the end of the trial (*P* < 0.001), which corresponded to higher daily weight gain (+17.7%), feed intake (+13.2%), final live weight (+17.3%), and lower feed conversion ratio (–3.2%) in the entire trial (Table 2).

By the end of the trial, 17 chickens had died (2.1% mortality) and 15 chickens (1.9%) were excluded from the trial because of health and welfare problems, without significant differences among experimental groups.

### Slaughter Results, Meat Quality, and Myopathy rate

No effect of dietary supplementation with Na-butyrate was observed on slaughter results, myopathy occurrence (Table 3), or meat quality traits (Table 4).

**Table 3:** Carcass traits (LS means) and myopathy rates (means) at commercial slaughtering at 42 d of age in chickens fed diets with different Na-butyrate supplementations and fat contents.

Variables	Na-butyrate (B)			Fat (F)		Sex (S)		*P*-value							RMSE
	B0	B150	B300	H	L	F	M	B	F	S	B × F	B × S	F × S	B × F x S	
Chickens, n	48	48	48	72	72	72	72								
Cold carcass weight, g CC	2135	2148	2145	2173	2112	1979	2306	0.880	<0.01	<0.001	0.910	0.952	0.466	0.936	138
Carcass yield, % LW	71.3	71.8	71.6	71.8	71.2	72.1	71.0	0.231	<0.05	<0.001	0.409	0.681	0.767	0.121	1.39
Dissected carcasses, n.	48	48	48	72	72	72	72								
Breast yield,^1^ % CC	40.5	40.4	40.4	40.7	40.2	41.4	39.6	0.977	0.091	<0.001	0.639	0.359	0.181	0.622	2.04
*Pectoralis major*, % CC	25.6	25.8	25.8	26.0	25.4	26.4	25.0	0.825	<0.05	<0.001	0.676	0.127	0.161	0.397	1.64
Wings, % CC	9.20	9.12	9.12	9.09	9.20	9.06	9.23	0.921	0.525	0.343	0.527	0.366	0.823	0.896	1.05
Thighs, % CC	16.7	16.9	16.9	16.8	16.9	16.3	17.4	0.589	0.752	<0.001	0.279	0.193	0.494	0.582	1.55
Drumsticks, % CC	13.9	14.0	13.8	13.9	13.9	13.5	14.3	0.678	0.678	<0.001	0.271	0.651	0.630	0.142	1.17
Hind legs, % CC	30.6	30.9	30.7	30.7	30.8	29.7	31.7	0.816	0.686	<0.001	0.243	0.328	0.501	0.172	2.41
Myopathy rates, %	48	48	48	72	72	72	72								
White striping	60.4	56.3	52.1	69.4	43.1	61.1	51.4	0.713	<0.01	0.240	0.890	0.590	0.237	0.089	-
Wooden breast^2^	22.9	27.1	22.9	31.9	16.7	11.1	37.5	0.860	<0.05	<0.001	0.807	0.050	0.698	0.070	-
Spaghetti meat	6.25	8.33	8.33	13.9	1.39	13.9	1.39	0.906	<0.01	<0.01	0.119	0.078	n.e	0.010	-

Abbreviations: RMSE, root mean square error where SEM is equal to RMSE/√n; B0, Control diet; B150, control diet supplemented with 150 mg/kg of Na-butyrate; B300, control diet supplemented with 300 mg/kg of Na-butyrate; H, Higher dietary fat content; L, Lower dietary fat content; F, females; M, males; n.e., not estimable.

1 With bone and skin.

2 Probability of the interaction Na-butyrate × sex, 20.8%, 4.17%, and 8.33% in females fed diet B0, B150, and B300, respectively; 25.0%, 50.0%, and 37.5% in males fed diet B0, B150, and B300, respectively.

**Table 4:** Rheological traits of *pectoralis major* and chemical composition of breast muscles (LS means) in broiler chickens fed diets with different Na-butyrate supplementations and fat contents.

Variables	Na-butyrate (B)			Fat (F)		Sex (S)		*P*-value							RMSE
	B0	B150	B300	H	L	F	M	B	F	S	B × F	B × S	F × S	B × F × S	
*Pectoralis major*, n	48	48	48	72	72	72	72								
pH	5.83	5.87	5.87	5.87	5.84	5.88	5.84	0.156	0.144	<0.05	0.718	0.666	0.596	0.893	0.12
L*	50.1	51.3	50.7	50.4	51.0	49.8	51.6	0.138	0.251	<0.001	0.858	0.804	0.263	0.525	2.96
a*	1.38	1.17	1.23	1.29	1.23	1.35	1.18	0.207	0.551	0.082	0.687	0.665	0.216	0.965	0.58
b*	13.7	13.8	13.5	13.5	13.8	13.5	13.8	0.617	0.120	0.078	0.831	0.299	0.329	0.527	1.29
Water losses,^1^ %	31.1	31.5	31.9	31.1	31.9	30.4	32.6	0.716	0.302	<0.01	0.440	0.367	0.701	0.812	4.91
Shear force, kg/g	3.38	3.33	3.39	3.35	3.39	3.28	3.46	0.917	0.732	0.179	0.624	0.532	0.837	0.113	0.78
Breast muscles, n	16	16	16	24	24	24	24								
Water, %	73.5	74.1	73.8	73.8	73.8	73.6	74.0	0.231	0.797	0.125	0.059	0.238	0.294	0.138	0.84
Crude protein, %	21.5	21.1	21.5	21.4	21.4	21.6	21.1	0.443	0.931	0.090	0.540	0.948	0.374	0.956	0.96
Ether extract, %	1.85	2.05	1.91	1.94	1.93	1.77	2.10	0.687	0.968	0.098	0.422	0.085	0.789	0.689	0.64
Ash, %	1.18	1.15	1.17	1.16	1.18	1.19	1.15	0.556	0.421	0.058	0.392	0.570	0.559	0.847	0.07

Abbreviations: RMSE, root mean square error where SEM is equal to RMSE/√n; B0, Control diet; B150, control diet supplemented with 150 mg/kg of Na-butyrate; B300, control diet supplemented with 300 mg/kg of Na-butyrate; H, Higher dietary fat content; L, Lower dietary fat content; F, females; M, males.

1 Thawing and cooking losses.

Chickens fed the LF diets had lighter carcass weight (−2.89%; *P* < 0.01) and lower carcass yield (−0.84%; *P* < 0.05) and *p. major* muscle proportion (−2.44%; *P* < 0.05) than did the chickens fed the HF diet (Table 3). Moreover, the former chickens tended to have a lower breast yield than the latter (*P* = 0.091) (Table 3). Chickens fed LF diets displayed lower rates of WS (–38%; *P* < 0.01), WB (–48%; *P* < 0.05), and SM (–90%; *P* < 0.01) than did chickens fed HF diets (Table 3), whereas no differences were observed for meat quality traits (Table 4).

Males had heavier carcasses (+16.5%; *P* < 0.001) and higher proportions of hind legs (+6.73%; *P* < 0.001), whereas displayed lower carcass (–1.52%; *P* < 0.001), breast (–4.35%; *P* < 0.0001), and *p. major* (–5.30%; *P* < 0.0001) yields than females (Table 3). Male chickens also had a lower SM (–12.5%; *P* < 0.01) and higher WB (+26.4%; *P* < 0.001) than female chickens (Table 4). Finally, males displayed a lower final pH (–0.68%; *P* < 0.05) and higher lightness (+3.61%; *P* < 0.001) and water loss (thawing and cooking) (+7.24%; *P* < 0.01) of breast meat than females (Table 4).

### Sensory Analysis of Breasts

Neither Na-butyrate supplementation nor dietary fat content affected the texture and flavor attributes of meat in the sensory test (Table 5). Regarding the effect of sex, fillets from males displayed higher juiciness (*P <* 0.001) and tended to have a sweeter flavor (*P* = 0.081) than fillets from females (Table 5).

**Table 5:** Sensory mean score (0–10 scale) of descriptive attributes of cooked breast fillets (*pectoralis major*) of broiler chickens fed diets with different Na-butyrate supplementations and fat contents.

Variables	Na-butyrate (B)			Fat (F)		Sex (S)		*P*-value							RMSE
	B0	B150	B300	H	L	F	M	B	F	S	B × F	B × S	F × S	B × F × S	
Chickens, n	16	16	16	24	24	24	24								
Texture															
Cohesiveness	3.85	4.15	3.90	3.99	3.94	3.86	4.08	0.585	0.862	0.384	0.138	0.281	0.360	0.917	2.01
Hardness	3.48	4.05	3.92	3.87	3.77	3.80	3.83	0.096	0.649	0.912	0.102	0.736	0.812	0.576	1.75
Juiciness	5.96	6.38	6.11	6.07	6.23	5.73	6.57	0.183	0.393	<0.001	0.192	0.535	0.251	0.405	1.50
Chewiness	4.59	4.80	5.00	4.89	4.70	4.70	4.89	0.295	0.376	0.395	0.308	0.682	0.888	0.481	1.70
Fibrosity	6.12	6.42	6.51	6.41	6.29	6.34	6.36	0.244	0.535	0.940	0.130	0.289	0.087	0.150	1.57
Flavor/taste															
Brothy	4.56	4.64	4.36	4.69	4.35	4.71	4.33	0.484	0.091	0.052	0.933	0.900	0.353	0.372	1.58
Chickeny/Meaty	5.97	6.01	5.93	6.01	5.94	6.10	5.85	0.898	0.619	0.097	0.370	0.314	0.964	0.309	1.12
Wet feathers	1.81	2.05	2.10	1.97	2.00	1.96	2.01	0.473	0.914	0.791	0.368	0.917	0.715	0.232	1.61
Sweet	6.06	5.80	6.04	6.07	5.87	5.81	6.12	0.397	0.234	0.081	0.601	0.887	0.704	0.546	1.37
Salty	4.66	4.80	4.69	4.68	4.75	4.76	4.67	0.726	0.676	0.562	0.768	0.888	0.811	0.490	1.26
Pleasantness	5.28	4.80	5.08	5.10	5.00	5.20	4.91	0.214	0.630	0.199	0.504	0.826	0.365	0.152	1.73

Abbreviations: RMSE, root mean square error where SEM is equal to RMSE/√n; B0, Control diet; B150, control diet supplemented with 150 mg/kg of Na-butyrate; B300, control diet supplemented with 300 mg/kg of Na-butyrate; H, Higher dietary fat content; L, Lower dietary fat content; F, females; M, males.

## DISCUSSION

### Effect of Butyrate Supplementation

Previous studies emphasized the positive effect of the dietary supplementation with butyric acid mainly associated with the effects on the gastrointestinal tract development and functionality, diet digestibility, and improved chicken immune response (Guilloteau et al., 2010; Bortoluzzi et al., 2018; Liu et al., 2019; Gao et al., 2024). Nevertheless, various authors (Leeson et al., 2005; Smulikowska et al., 2009; Pascual et al., 2020; de-Cara et al., 2023) did not observe a growth-promoting response or reported reduced growth as a consequence of the dietary supplementation with butyric acid or its salts when chickens were reared in an environment with low pathogens or favorable state of health, similar to the conditions of the present trial. More recently, Melaku et al. (2024) reported the positive effect of a new buffer salt-protected Na-butyrate on growth performance, by improving intestinal histomorphology, barrier function, antioxidative capacity, and the microbiota community of broilers, even in unchallenged conditions. However, butyrate supplementation is expected to be more effective in young chicks when it plays a regulatory role in gut cells by increasing the absorption surface and nutrient absorption capacity (Bedford and Gond, 2018; Lan et al., 2020). Consistent with previous findings (Hu and Guo, 2007; Gao et al., 2022; de-Cara et al., 2023), the present study found a favorable effect of butyrate supplementation on the performance of chickens during the first phase (0–21 d), which, however, disappeared by the end of the trial.

An increase in carcass weight and breast yield as a consequence of butyrate supplementation has been reported in some studies (Leeson et al., 2005; Panda et al., 2009; Namkung et al., 2011; Mátis et al., 2019), whereas others did not observe any changes (Pascual et al., 2020; Yang et al., 2022; de-Cara et al., 2023). In fact, butyric acid is expected to play a role in different pathways, such as lipolysis, lipogenesis, and gluconeogenesis (Heimann et al., 2015), as well as in regulating cell growth and differentiation programming (Liang et al., 2010; Mali et al. 2010; Berni Canani et al., 2012), which has been associated with effects on muscle fibre in pigs (Duan et al., 2018) and meat quality in broiler chickens (Wu et al., 2020). However, both in our study and previous studies (Zhang et al., 2011; Gomathi et al., 2018; Pascual et al., 2020), meat quality was not affected by butyrate supplementation in terms of rheological traits, whereas other authors have reported darker meat, higher pH (Gao et al., 2022), and decreased saturated fatty acids content in the breast meat of chickens exposed to stress (Zhang et al., 2011). To the best of our knowledge, no previous study has evaluated the effect of Na-butyrate supplementation on the sensory characteristics of cooked meat as measured by trained panelists. However, major changes in meat texture and flavor traits can be expected as a consequence of differences in water retention properties, fat content, and quality, which were not observed in the present study between chickens supplemented with butyrate, where differences in meat taste are determined by free amino acids, which were not analyzed in the present study (Pérez-Santaescolástica et al., 2018).

The hypothesis that the inclusion of butyrate could mitigate myopathies was not confirmed in the present study. Previously, Pascual et al. (2020) observed a reduction in the SM rate in females fed 0.05% microencapsulated Na-butyrate compared to those fed the control diet, corroborating other studies that described muscle responses related to oxidative and hypoxic metabolism in pigs and broiler chickens as a consequence of dietary supplementation with butyrate (Duan et al., 2018; Wu et al., 2020). Dietary manipulation of nutrients that can play a role on the oxygen homeostasis (i.e., inositol, phytase, and guanidine acetic acid) has been demonstrated to be almost always successful in reducing the occurrence of myopathies in broiler chickens compared to the use of feed additives acting directly as antioxidants or feed additives with other metabolic roles (Trocino et al., 2023). All myopathies involve oxidative stress and hypoxia, which trigger muscle degeneration (Soglia et al., 2021). All cells produce reactive oxygen species; their generation and the protection provided by antioxidants should be balanced to avoid muscle degeneration, mitochondrial dysfunction, inflammation, and insufficient muscle regeneration directly linked to oxidative stress (Forman and Zhang, 2021; Mosca et al., 2021). Tang et al. (2022) reported that dietary supplementation with Na-butyrate activated antioxidant pathways, improved antioxidant capacity, and reduced oxidative stress in rats fed high-fat diets by reducing reactive oxygen species levels and interfering with gene expression regulation, thereby improving mitochondrial function and increasing insulin secretion and muscle insulin sensitivity. Other authors have hypothesized that dietary supplementation with Na-butyrate has an antioxidant effect in the gut (Wu et al., 2018) and breast muscles (Wu et al., 2020). Nevertheless, owing to inconsistencies among the results, further investigations are required to definitively state if and how dietary supplementation with Na-butyrate might play a role in the occurrence of myopathies and its possible mitigation.

### Effect of Dietary Fat Reduction

Fast growth and high breast yield, which characterize the selected commercial genotypes used in poultry production, have also been identified as triggering causes of myopathy occurrence (Petracci et al., 2019; Soglia et al., 2021). Several studies have identified high live weight (Che et al., 2022) and breast yield as risk factors for WB and WS development and occurrence (Lake and Abasht, 2020; Bordignon et al., 2022). Reviewing studies investigating nutritional and feeding strategies for controlling myopathy occurrence, Trocino et al. (2023) found a significant correlation between the effects on growth (final body weight and daily weight gain) and myopathy occurrence (across WS, WB, and SM). Thus, several studies have investigated different nutritional and feeding strategies intended to manipulate the growth trajectory and breast muscle development to mitigate myopathy occurrence (Bailey, 2023; Trocino et al., 2023), and low-to-moderate hereditability and a high contribution of environmental and management factors (>65% for WS and >95% for WB) to myopathy occurrence have been highlighted (Bailey et al., 2015; Bailey, 2023).

Most of these studies used qualitative (e.g., macronutrients, i.e., protein and energy, as well as amino acids) and quantitative (using feed restriction) nutrient manipulation and allocation, which successfully controlled myopathy occurrence and was affected by the stage of growth at which nutrient allocation was applied, as well as the duration of the manipulation period (Trocino et al., 2023). Under the conditions of the present study, the reduction in dietary energy levels by reducing fat content mitigated the occurrence of all myopathies (WS, WB, and SM). Kuttappan et al. (2012a) reported a reduction in the occurrence of myopathy by lowering the energy value of diets, whereas Meloche et al. (2018a) found a reduction in the severity of WS and WB by reducing broiler feed intake. In contrast, Trocino et al. (2015) found that quantitative feed restriction from 13 to 21 d of age increased WS occurrence at the end of the trial compared with *ad libitum* feeding during the entire trial. The success of these strategies depends on the rate and duration of restriction as well as the duration of the refeeding phase. The dilution or limitation of feed and nutrient intake impairs growth (Zhai et al., 2014; Gopinger et al., 2017; Meloche et al., 2018a,b). In the present study, owing to the young age of chickens and imperfect chemical regulation of appetite, feed intake in chickens fed diets with different fat levels was the same as that of those fed diets with a lower fat level, which slowed down their growth owing to the lower daily energy intake. Meloche et al. (2018b) reported that a difference of 10% in dietary energy levels was necessary to produce differences in feed intake among groups, whereas under our conditions, the difference in energy content between the high- and low-fat diets was only 1.7% in the grower diets (calculated ME: 3,107 vs. 3,056 kcal/kg) and 3.3% in the finisher diets (ME: 3,215 vs. 3,113 kcal/kg). Similarly, Pires et al. (2022) did not report significant differences in feed intake when feeding chickens with dietary energy decreased by 90–100 kcal/kg. In the present study, the lower final live weight of chickens fed diets with a lower level of fat was associated with decreased carcass weight and yield, whereas the proportions of the main carcass cuts were not affected, which is consistent with the results of previous studies (Abudabos et al., 2014; Infante-Rodríguez et al., 2016; Gopinger et al., 2017). However, the extent of the reduction in myopathy occurrence was significantly higher than the reduction in growth performance and carcass traits, and the balance between the 2 results must be carefully evaluated from an economic perspective at a farm level.

Qualitative feed restrictions can also affect the meat quality when a high restriction rate is applied. When feeding chickens low-energy diets, the fat content of carcasses and meat can increase owing to an unbalanced use of dietary nutrients (Chang et al., 2022) and excessive fat deposition at the muscle level (Boekholt et al., 1994; Albuquerque et al., 2003; Ahiwe et al., 2018; Meloche et al., 2018b). In contrast, the protein content of meat under nonextreme dietary nutrient restriction regimes is usually rather stable, as reported by Infante-Rodríguez et al. (2016) and consistent with our results.

The rheological traits of meat can be affected by quantitative and qualitative nutrient restriction due to changes in the energy metabolism of the muscle, depending on the rate and duration of the restriction. Lipiński et al. (2019) observed an increase in pH and lightness, and a reduction in the redness index in the breasts of chickens fed low-energy diets, in addition to changes in water-holding capacity. However, the results of the present study are consistent with those of Arshad et al. (2020), who did not observe changes in meat pH or lightness in chickens fed diets with differences of 100 kcal ME/kg. Thus, no studies have associated meat sensory traits with changes in the energy of the diet; however, high amounts of intramuscular fat are known to influence meat flavor (Kanokruangrong et al., 2019). Especially for chicken meat, tenderness and juiciness, rather than aroma or color, can have greater repercussions on the overall liking of consumers (Kanokruangrong et al., 2019) and can be related to the water-holding capacity of meat. Under our study conditions, without changes in meat pH, water loss (thawing and cooking) was not influenced by the energy level of the diet; thus, the sensory properties of meat were not affected.

### Effect of Sex

The results of the present trial confirmed that compared with female chickens, male broiler chickens have a high growth rate (Trocino et al., 2015; Cygan-Szczegielniak et al., 2019; Pascual et al., 2020), low breast and *p. major* muscle yields, and high leg yield (López et al., 2011; Huerta et al., 2023). The results of the present study also confirmed main differences in myopathy occurrence, i.e., a higher rate of WB in males and a higher rate of SM in females (Trocino et al., 2015; Pascual et al., 2020; Bordignon et al., 2022; Novoa et al., 2022; Bošković Cabrol et al., 2024). We also confirmed that WS occurrence is similar in the 2 sexes as reported in previous studies (Castilho et al., 2021; Bordignon et al., 2022; Bošković Cabrol et al., 2024).

In contrast, considering the main rheological and chemical traits, meat quality was not different between sexes as previously reported for the genotype (Bošković Cabrol et al., 2024). A previous study has described some differences in meat fatty acid composition between females and males, which were not investigated in the present study and could be related to sex differences in metabolism (Domínguez et al., 2014). In detail, when comparing males with females, higher rates of polyunsaturated fatty acids owing to higher linoleic and γ-linolenic acids have been reported in the meat of fast- (Stanišić et al., 2023; Bošković Cabrol et al., 2024) and slow-growing chickens (Cerolini et al., 2019; Bongiorno et al. 2022).

Regarding the sensory attributes of cooked meat, the high juiciness measured in the meat of males compared with that of females was not consistent with the low meat pH and high thawing and cooking losses of the meat of the former compared with that of the latter. In contrast, the higher sweet flavor recorded in the present study for the meat obtained from males than for that from females has been described previously (Bošković Cabrol et al., 2024) which was attributed to possible differences in amino acid, mineral, and fatty acid composition in meat between sexes. However, Stęczny and Kokoszynski (2019) did not find any differences in sensory attributes of meat between the sexes in 42-day-old Ross 308 broiler chickens.

## CONCLUSIONS

The cost-benefit balance of modulating the growth trajectory by dietary energy in broiler chickens must be evaluated in terms of animal welfare, consumer perception, and economic results, where even a moderate reduction of dietary energy can reduce myopathy occurrences considerably with marginal impairment of growth performance. However, further studies are necessary to determine the effect of Na-butyrate dietary supplementation on growth performance, carcass and meat quality, where its use as an additive to improve gut health remains a valid option.

## DISCLOSURES

The authors declare that they have no known competing financial interests or personal relationships that could have appeared to influence the work reported in this paper.
